# Exploring the Diversity and Regulation of Apocarotenoid Metabolic Pathways in Plants

**DOI:** 10.3389/fpls.2021.787049

**Published:** 2021-12-10

**Authors:** Xiongjie Zheng, Yu Yang, Salim Al-Babili

**Affiliations:** The BioActives Lab, Center for Desert Agriculture (CDA), Biological and Environment Science and Engineering (BESE), King Abdullah University of Science and Technology, Thuwal, Saudi Arabia

**Keywords:** carotenoids, apocarotenoids, pigments, volatiles, natural variation, molecular regulation

## Abstract

In plants, carotenoids are subjected to enzyme-catalyzed oxidative cleavage reactions as well as to non-enzymatic degradation processes, which produce various carbonyl products called apocarotenoids. These conversions control carotenoid content in different tissues and give rise to apocarotenoid hormones and signaling molecules, which play important roles in plant growth and development, response to environmental stimuli, and in interactions with surrounding organisms. In addition, carotenoid cleavage gives rise to apocarotenoid pigments and volatiles that contribute to the color and flavor of many flowers and several fruits. Some apocarotenoid pigments, such as crocins and bixin, are widely utilized as colorants and additives in food and cosmetic industry and also have health-promoting properties. Considering the importance of this class of metabolites, investigation of apocarotenoid diversity and regulation has increasingly attracted the attention of plant biologists. Here, we provide an update on the plant apocarotenoid biosynthetic pathway, especially highlighting the diversity of the enzyme carotenoid cleavage dioxygenase 4 (CCD4) from different plant species with respect to substrate specificity and regioselectivity, which contribute to the formation of diverse apocarotenoid volatiles and pigments. In addition, we summarize the regulation of apocarotenoid metabolic pathway at transcriptional, post-translational, and epigenetic levels. Finally, we describe inter- and intraspecies variation in apocarotenoid production observed in many important horticulture crops and depict recent progress in elucidating the genetic basis of the natural variation in the composition and amount of apocarotenoids. We propose that the illustration of biochemical, genetic, and evolutionary background of apocarotenoid diversity would not only accelerate the discovery of unknown biosynthetic and regulatory genes of bioactive apocarotenoids but also enable the identification of genetic variation of causal genes for marker-assisted improvement of aroma and color of fruits and vegetables and CRISPR-based next-generation metabolic engineering of high-value apocarotenoids.

## Introduction

Carotenoids are lipophilic isoprenoid molecules consisting of a polyene backbone that generally contains 3–11 conjugated double bounds. They can be synthesized *de novo* by plants, algae, photosynthetic bacteria, and many nonphotosynthetic microorganisms (Hirschberg, 2001; Fraser and Bramley, 2004; DellaPenna and Pogson, 2006; Moise et al., 2014; Rodriguez-Concepcion et al., 2018; Zheng et al., 2020a). Humans cannot build carotenoids *de novo*, but need them as important antioxidants and as essential source of provitamin A, particularly if animal-derived food is not available (Fraser and Bramley, 2004; DellaPenna and Pogson, 2006; Giuliano, 2014; Nisar et al., 2015; Zheng et al., 2020b).

In many crops and other plant species, carotenoids confer their vivid yellow to red colors to fruits, flowers, tubers, and seeds (Yuan et al., 2015; Giuliano, 2017), which attract animal pollinators and distributors and are important feature for consumers, which indicate the quality of plant products and, hence, promote their marketability. More importantly, carotenoids are essential for plant photosynthesis, as they protect the photosynthetic apparatus from photooxidative damage and contribute to light-harvesting (Hashimoto et al., 2016; Rodriguez-Concepcion et al., 2018; Moreno et al., 2021). The conjugated double bonds make carotenoids themselves susceptible to photooxidation and other unspecific oxidative breakdown processes, such as lipoxygenase mediated co-oxidation, as well as to targeted, enzyme-catalyzed cleavage of certain double bonds (Zheng et al., 2020a; Moreno et al., 2021). The term “apocarotenoid” is used to define the cleavage products arising from these processes. The large number of double bonds in carotenoid backbone and of different carotenoids leads to a plentitude of apocarotenoids with various physico-chemical properties and biological functions. The compound family of apocarotenoids includes precursors of important phytohormones, that is, abscisic acid (ABA) and strigolactones (SLs), signaling molecules involved in plant growth, development, and resistance against pathogens and herbivores (Jia et al., 2018; Felemban et al., 2019; Watkins and Pogson, 2020; Wang et al., 2020b), as well as apocarotenoid pigments and volatiles responsible for color and aroma of fruits, flowers, or vegetables (Liang et al., 2021; Zheng et al., 2021).

Plant apocarotenoids and their derivatives have also important functions in promoting human health (Rodriguez-Concepcion et al., 2018). Humans contain two carotenoid cleavage enzymes, i.e., β-carotene cleavage oxygenase 1 and 2 (BCO1 and 2), which convert some carotenoids into a restricted set of apocarotenoids, including vitamin A (Harrison, 2005; Von Lintig, 2010); however, they cannot produce many health-promoting apocarotenoids, such as crocins and safranal (Frusciante et al., 2014; Ahrazem et al., 2016b). Given the importance of apocarotenoids for plants and humans, numerous studies have been performed on investigating the biological functions of apocarotenoids, elucidating their biosynthetic pathways and the regulation of their formation. Naturally occurring inter- or intraspecies apocarotenoid variation was observed in some important crops, such as citrus, tomato, and *Capsicum* species (Gao et al., 2019; Zheng et al., 2019; Zoccali et al., 2021), which would help to decipher apocarotenoid biosynthesis and its regulation, and genetic control of apocarotenoid diversity. Nowadays, the advancements in global profiling strategies, such as genomics, transcriptomics, and metabolomics, and multi-omics approaches have been very helpful for investigating the variation of apocarotenoid levels as well as the underlying genetic mechanisms. Several recent reviews cover the biosynthesis of apocarotenoid-derived hormones and signaling molecules and their roles in plant development and growth (Felemban et al., 2019; Fiorilli et al., 2019; Wang et al., 2020b; Moreno et al., 2021). In this review, we will mainly focus on the advances that have recently been made in understanding the regulation, metabolism, and genetic mechanism underlying apocarotenoid diversity, particularly apocarotenoid pigments and volatiles. We also depict the possible impact of understanding the mechanisms behind apocarotenoids diversity on accelerating molecular breeding and CRISPR-based next-generation metabolic engineering of apocarotenoids for crop improvement.

## The Significance and Function of Apocarotenoids

Non-enzymatic and enzymatic oxidative cleavage of carotenoids and further metabolism of thereby arising apocarotenoids lead to various biologically important plant metabolites, including phytohormones, pigments, volatiles, and signaling molecules (Table 1). Due to their important roles in many physiological, developmental processes, and plant-biotic environment interactions, the phytohormones ABA and SLs are best-known examples for biologically important plant apocarotenoids. ABA is widely distributed in nature and is common in cyanobacteria, algae, fungi, plants as well as in animals (Wang et al., 2020b). It plays important roles in many physiological and developmental processes, including the establishment of seed dormancy, root, and shoot growth and regulation of stomatal closure, and is a major component in plant abiotic and biotic stress response (Chen et al., 2020; Moreno et al., 2021). SLs were originally discovered as the host-derived rhizospheric chemical signal that induces seed germination in root parasitic plants, such as *Striga hermonthica*, which represent a major threat to global food security (Xie et al., 2010; Jamil et al., 2021). Later studies on arbuscular mycorrhization revealed the role of SLs as the plant-derived signal that induces hyphal branching in AM-fungi, which paves the way for host colonization (Cook et al., 1966; Lanfranco et al., 2018; Wang et al., 2020b). Within plants, SLs inhibit shoot branching and the formation of adventitious roots, stimulate internodes elongation, and increase stem thickness (Al-Babili and Bouwmeester, 2015; Moreno et al., 2021). Furthermore, SLs are involved in leaf and floral organ senescence, as well as in biotic and abiotic stress response (Al-Babili and Bouwmeester, 2015; Decker et al., 2017; Wang et al., 2020b). Besides these two apocarotenoid-derived phytohormones, recent studies on the formation and biological functions of apocarotenoids unraveled new carotenoid-derived plant signaling molecules and growth regulators. For example, Wang et al. (2019a) identified a new growth regulator named zaxinone, which promotes rice growth and modulates its hormone homeostasis. Exogenous application of zaxinone as well as of zaxinone mimics increased the growth of crown roots of rice seedlings and alleviated *Striga*-infestation in a *Striga*-susceptible rice cultivar, by reducing SL content and release (Wang et al., 2019a, 2020a), demonstrating its application potential for improving rice growth and combating root parasitic weeds. Very recently, Wang et al. (2021) also demonstrated that increased sugar uptake and metabolism is likely the major reason for growth-promoting activities of zaxinone in rice and that zaxinone effect on root cell division activity in root meristem and on the number of cortex cell layers is likely caused by modulating cytokinin content. The enzyme involved in zaxinone formation in rice, the carotenoid cleavage dioxygenase (CCD) Zaxinone synthase, is common in mycorrhizal plants, but absent in those, such as *Arabidopsis thaliana*, that do not build this symbiosis (Wang et al., 2019a). In contrast to rice, a recent study showed that zaxinone application to hydroponically grown *Arabidopsis* seedlings increased SL and ABA content in roots and did not improve growth. This result suggests that the activities and functions of zaxinone may differ between plant species and are likely coupled to their ability to establish arbuscular mycorrhizal fungi (AMF) symbiosis (Wang et al., 2019a; Ablazov et al., 2020; Moreno et al., 2021).

**Table 1:** Biosynthesis and biological functions of plant apocarotenoids and their derivatives.

Apocarotenoid	CCD enzymes involved	Biological functions	References
Strigolactones	CCD7; CCD8	Phytohormones involved in different developmental processes and rhizospheric signaling molecules inducing seed germination of root parasitic plants and hyphal branching of arbuscular mycorrhizal (AM) fungi. The latter is needed for establishing the AM symbiosis.	Alder et al., 2012; Zhang et al., 2014				
Carlactone	CCD7; CCD8	The central intermediate in of strigolactone biosynthesis.	Alder et al., 2012; Bruno et al., 2014				
3-Hydroxy-carlactone	CCD7; CCD8	A precursor of yet-unidentified strigolactones.	Baz et al., 2018; Yoneyama et al., 2020				
Abscisic acids	NCEDs	A phytohormone involved in plant abiotic and biotic stress response as well as in many developmental processes including seed dormancy.	Tan et al., 2003; Moreno et al., 2021				
Zaxinone	ZAS (CCD10)	A natural growth regulator that promotes rice root growth and is required for normal rice growth and development. It is involved in mycorrhization and regulates SL and ABA biosynthesis in *Arabidopsis*	Wang et al., 2019a, 2021; Ablazov et al., 2020; Zhong et al., 2020				
Anchorene	Unknown	An apocarotenoid dialdehyde and natural plant metabolite involved in the formation of Arabidopsis anchor roots.	Jia et al., 2019				
Iso-anchorene	Unknown	An isomer of anchorene, which inhibits primary root growth in Arabidopsis.	Jia et al., 2021a				
β-Cyclocitral	Citrus CCD4b	A cyclic volatile apocarotenoid with a tobacco-like or grassy flavor. It acts as a signaling molecule regulating oxidative stress response, promotes Arabidopsis root growth, and induces plant resistance against *Spodoptera littoralis* as an herbivore-triggered signal;	Ramel et al., 2012; Dickinson et al., 2019; Gao et al., 2019; Mitra et al., 2021; Zheng et al., 2021				
β-Ionone	CCD1, CCD4, or CCD7	A cyclic volatile apocarotenoid with a violet-like or fruity aroma. It attracts pollinators and seed dispersers and shows a strong repellent effect toward both the spider mite and flea beetle.	Simkin et al., 2004a; Cáceres et al., 2016; Liang et al., 2021				
α-Ionone	CCD1/CCD4	A cyclic volatile apocarotenoid with a violet-like or fruity aroma. It attracts pollinators and seed dispersers and induces tomato resistance against western flower thrips;	Murata et al., 2020; Liang et al., 2021
			
β-Damascenone	Unknown	A cyclic volatile apocarotenoid with a honey-like, fruity aroma, and a super low odor threshold for perception. It contributes to the aroma of fruits and flowers.	Shi et al., 2020; Liang et al., 2021				
6-Methyl-5-hepten-2-one	CCD1/4	A linear volatile apocarotenoid and an important aroma component of fruits and flowers	Shi et al., 2020; Liang et al., 2021				
Geranylace-Tone	CCD1/4	A linear volatile apocarotenoid and an important aroma component of fruits and flowers	Simkin et al., 2004a; Lashbrooke et al., 2013				
Loliolide or (−)-Loliolide	Unknown	An endogenous regulatory metabolite that mediates plant defense response to herbivores and enhances production of allelochemicals in the barnyardgrass-rice allelopathic interactions.	Murata et al., 2019; Li et al., 2020				
Bixin	*Bixa* CCD4	A di-carboxylic monomethyl ester apocarotenoid that confers red color to *Bixa orellana* seeds. It is used in food and cosmetic industry and shows anti-inflammatory and antinociceptive activities.	Bouvier et al., 2003; Pacheco et al., 2019				
Crocetin	*Crocus* CCD2, *Gardenia* CCD4a *Buddleja* CCD4.1/3	A natural apocarotenoid dicarboxylic acid mainly found in red stigmas of crocus flowers and gardenia fruits. It shows significant antitumorigenic effects in cell culture systems and animal models.	Gutheil et al., 2012; Frusciante et al., 2014; Ahrazem et al., 2016b, 2017				
Crocins	*Crocus* CCD2, *Gardenia* CCD4a *Buddleja* CCD4.1/3	Natural water-soluble apocarotenoids that consist of a group of crocetin glycosides. They are mainly found in red stigmas of crocus flowers and gardenia fruit. They have different pharmacological effects, such as anti-inflammatory, antiaging, analgesic, and neuroprotective;	Frusciante et al., 2014; Ahrazem et al., 2016b, 2017; Bukhari et al., 2018				
Picrocrocin	*Crocus* CCD2	A β-D-glucoside of 3-OH-β-cyclocitral. It is the precursor of safranal and responsible for the bitter taste of stigmas of crocus. It has pharmacological effects, such as reduction of the proliferation of human malignant melanoma, and is used in medical, food, and cosmetics industry.	Diretto et al., 2019; Martí et al., 2020				
Safranal	*Crocus* CCD2	An apocarotenoid with pungent aroma and a major volatile component of crocus stigma.	Rezaee and Hosseinzadeh, 2013; Diretto et al., 2019				
β-Citraurin	*Citrus* CCD4b	A C_30_ red apocarotenoid pigment responsible the red peel of citrus fruit.	Ma et al., 2013; Rodrigo et al., 2013; Zheng et al., 2019				
β-Citraurinene	*Citrus* CCD4b	An C_30_ apocarotenoid pigment mainly found in red peel of citrus fruit.	Zheng et al., 2019

Oxidative cleavage of carotenoids at more than one double bond results in the production of various dialdehyde products. Jia et al. (2021a) identified a carotenoid-derived C_10_-dialdehyde that was named anchorene, which specifically promoted the growth and development of Arabidopsis anchor roots. This type of roots emerges from the collet region, the root–hypocotyl junction, through modulating auxin homeostasis. The identity of anchorene as a natural carotenoid-derived metabolite was confirmed using mutants affected in carotenoid biosynthesis and chemical inhibitors of carotenoid biosynthesis and by establishing a LC-MS-based system for isolating and detecting of carotenoid-derived dialdehydes (Jia et al., 2019; Mi et al., 2019). The discovery of anchorene unraveled, for the first time, a biological function of carotenoid-derived dialdehydes in plant development.

β-Cyclocitral (β-CC) is a volatile apocarotenoid derived from the oxidation of carotenoids (e.g., β-carotene), which is widely present in nature and is common in various organisms ranging from cyanobacteria and fungi to plants. It was shown that β-CC is a retrograde signaling molecule regulating the expression of oxidative stress-responsive genes, resulting in acclimation of plants to high-light conditions (Ramel et al., 2012). β-cyclocitric acid (β-CCA), a direct oxidation product of β-CC, is also a stress signal that enhanced drought tolerance likely through a signaling pathway different from that of β-CC (D'alessandro et al., 2019). Because of its water-soluble character, β-CCA is more suitable for agricultural application and can be more easily used to increase drought tolerance of crops through irrigation or by spraying, compared with its precursor β-CC (Havaux, 2020). In addition to its role in oxidative stress response, β-CC is a root growth regulator. Exogenous application of β-CC promoted the growth of primary roots in Arabidopsis, tomato, and rice. It was also shown that β-CC alleviates the inhibitory effect of salt on the growth of rice roots (Dickinson et al., 2019). A recent study showed that β-CC induces plant resistance against *Spodoptera littoralis*, as an herbivore-triggered signal, and downregulates the methylerythritol-4-phosphate (MEP) pathway flux, which is responsible for plastid isoprenoid biosynthesis, through direct inhibition of 1-deoxy-*D*-xylulose-5-phosphate synthase (DXS), a rate-limiting enzyme of the MEP pathway (Mitra et al., 2021). Other volatile apocarotenoids are also involved in herbivore resistance or response. For example, β-ionone is released as herbivores-induced defense volatile in canola, *Brassica napus*, and shows a strong repellent effect toward both the spider mite and flea beetle (Cáceres et al., 2016). α-Ionone, a structural isomer of β-ionone, induces jasmonic acid (JA) independent resistance to western flower thrips in tomato and Arabidopsis, by reducing the survival rate of western flower thrips without exhibiting direct insecticidal activity (Murata et al., 2020). Loliolide, a C_11_-terpene lactone, functions as an endogenous signaling metabolite that induces resistance to multiple herbivores, such as the two-spotted spider mite, common cutworm, and western flower thrips, possibly independent of JA signaling (Murata et al., 2019). Application of loliolide to tomato leaves reduced the survival rate of the two-spotted spider mite and larvae of the common cutworm, without exhibiting direct toxic activity against these herbivores (Murata et al., 2019). Moreover, loliolide [also referred to as (–)-loliolide] was found to act as a soil-borne signaling molecule that enhances the production of allelochemical, such as momilactone B and tricin, to coordinate barnyardgrass-rice allelopathic interactions (Kong et al., 2018; Li et al., 2020).

Besides their biological functions within plants, many apocarotenoid volatiles are recognized as important aroma components of different flowers and fruits of horticultural plants (Shi et al., 2020). For instance, linear volatile apocarotenoids, such as geranyl acetone, 6-methyl-5-hepten-2-one (MHO), and geranial, were identified as important aroma components in fruits of papaya, citrus, and blackberry (González-Mas et al., 2011; Jing et al., 2015; Reidel et al., 2016). Cyclic apocarotenoids, such as β-CC, β-ionone, and β-damascenone, have lower odor thresholds and stronger impacts on people’s aroma perception, compared with linear volatiles (Goff and Klee, 2006). Therefore, cyclic apocarotenoids are recognized as more important volatiles with respect to the aromatic odor of fruits and flowers (Shi et al., 2020). The C_13_ cyclic β-damascenone has an attractive honey-like, floral, and fruity flavor and shows a very low odor threshold of 2 ng/L on people’s aroma perception, hence, it has been frequently used as flavoring ingredient in fragrance industry (Liang et al., 2021). The cyclic volatiles α- and β-ionone have violet-like, woody, and fruity odor, while β-CC has a grassy, woody, tobacco-like odor. Both of them are widely used as flavor molecules in fragrances. In addition, these apocarotenoids volatiles also function as effective attractants for pollinators and seed dispersers (Liang et al., 2021).

Apart from being aroma compounds, apocarotenoids can contribute to the color of flowers and fruits and act as antioxidants. Indeed, some long-chain apocarotenoids and their derivatives are used as high-value food colorants, cosmetic agents, and antioxidants and have an important impact in promoting human health. β-citraurin (3-hydroxy-β-apo-8′-carotenal) and β-citraurinene are two major C_30_ apocarotenoid pigments found in *Citrus*, and their hyper-accumulation is responsible for the attractive red coloration of fruit peel in some citrus species (Zheng et al., 2019). Bixin (C_25_) is a further natural apocarotenoid pigment that confers red color to *B. orellana* seeds. It has been widely extracted from natural sources and used in food and cosmetic industry as a color additive (Giuliano et al., 2003b). Furthermore, Pacheco et al. (2019) demonstrated anti-inflammatory and antinociceptive activities of bixin, which were shown to be caused by reduction of neutrophil migration in simulated wound tests in rats. The proposed biosynthetic pathway of bixin is supposed to start with the cleavage of all-*trans*-lycopene into bixin aldehyde, followed by three sequential conversion steps catalyzed by the bixin aldehyde dehydrogenase and nor-bixin carboxyl methyltransferase (Bouvier et al., 2003). Saffron is one of the oldest natural food additives and worldwide the most expensive spice. It corresponds to the stigma of saffron flowers, which accumulates the 3-OH-β-cyclocitral glycoside picrocrocin, the C_10_ volatile apocarotenoid safranal, and the C_20_ pigment crocetin and its glycosides crocins. Crocetins and crocins are responsible for the red color, while picrocrocin and safranal give rise for the bitter taste and pungent aroma of saffron (Frusciante et al., 2014). These apocarotenoids are beneficial to health and are commonly used in food and cosmetics in many countries. Saffron apocarotenoids were also shown to have several pharmacological effects, such as anti-inflammatory, antiaging, analgesic, and neuroprotective (Bukhari et al., 2018).

## The Metabolic Background of Plant Apocarotenoids Structural Diversity

### Various Carotenoid Cleavage Dioxygenases Mediate Site-Specific Oxidative Tailoring of Carotenoids, Forming a Diverse Set of Apocarotenoids

**Figure 1:**
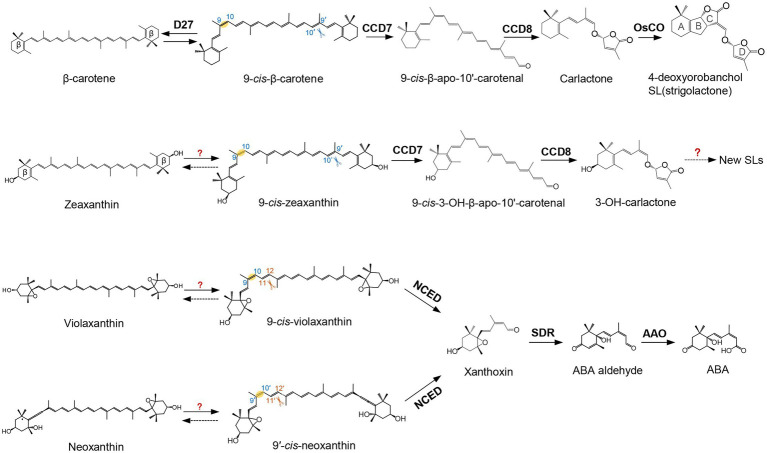
Biosynthetic pathway of carotenoid-derived phytohormones. Names of carotenoids and apocarotenoids are shown in black. Names of carotenoid cleavage dioxygenases and apocarotenoid modifying enzymes are depicted in black boldface. D27 catalyzes the reversible isomerization of all-*trans*-β-carotene into 9-*cis*-β-carotene. 9-*cis*-β-carotene is then converted into strigolactones (SLs; 4-deoxyorobanchol is depicted as an example) by CCD7, CCD8, and CYP enzymes, such as the rice carlactone oxidase (OsCO), a homolog of the Arabidopsis MAX1. Violaxanthin and neoxanthin can be converted into 9-*cis*-violaxanthin and 9'-*cis*-neoxanthin, respectively. Nine-*cis*-Epoxycarotenoid Dioxygenases (NCEDs) cleave these two *cis* epoxy-xanthophylls to yield xanthoxin. Xanthoxin is then converted into abscisic acid (ABA) by short-chain dehydrogenase (SDR reductase) and abscisic aldehyde oxidase (AAO). Scissors indicate the double bond positions cleaved by carotenoid cleavage dioxygenase (CCD) enzymes.

In plants, the enzymatic cleavage of carotenoids at specific positions is generally catalyzed by CCDs, non-heme iron-dependent enzymes present in plants, animals, bacteria, and fungi (Giuliano et al., 2003a; Sui et al., 2013; Ahrazem et al., 2016a). In the last decades, different types of plant CCDs with distinct features have been identified and investigated. Arabidopsis contains five types of CCD enzymes, known as CCD1, CCD4, CCD7, CCD8, and 9-*cis*-epoxycarotenoid dioxygenase (NCED). There are five NCEDs in Arabidopsis, NCED2, NCED3, NCED5, NCED6, and NCED9, while the other types are represented by a single enzyme each (Auldridge et al., 2006; Walter and Strack, 2011). The five NCEDs catalyze the oxidative cleavage of 9-*cis*-violaxanthin and/or 9′-*cis*-neoxanthin to produce xanthoxin, the precursor of ABA (Figure 1; Schwartz et al., 1997; Tan et al., 2003; Al-Babili and Bouwmeester, 2015; Jia et al., 2018). This apocarotenoid product is exported from plastids, the site of carotenoid biosynthesis and most of plant cleavage enzymes, to the cytosol and converted to abscisic aldehyde by the enzyme short-chain dehydrogenase (SDR; González-Guzmán et al., 2002). The arising abscisic aldehyde is then oxidized by a molybdenum-dependent aldehyde oxidase (AAO) to produce ABA (Seo et al., 2000). Very recently, Jia et al. (2021b) found that β-apo-11-carotenoids (C_15_), i.e., β-apo-11-carotenal, 9-*cis*-β-apo-11-carotenal, 3-OH-β-apo-11-carotenal, and 9-*cis*-3-OH-β-apo-11-carotenal, exert ABA-like biological functions in maintaining seed dormancy and inducing the expression of ABA-responsive genes. Further feeding experiments with labeled apocarotenoids, combined with analysis of physiological and transcriptional responses, showed that plants can synthesize ABA from C_15_ β-apo-11-carotenoids, which represents an alternative, zeaxanthin epoxidase-independent ABA biosynthetic pathway (Jia et al., 2021b). The genes involved in the hydroxylation, isomerization, and oxidation the C_15_ β-apo-11-carotenoids in this new ABA biosynthesis pathway remain elusive, which would be important for future exploration of the biological significance of this route.

The other four CCDs of Arabidopsis, CCD1, CCD4, CCD7, and CCD8 show different cleavage activities and biological functions (Zheng et al., 2020a). CCD7 and CCD8 are involved in SL biosynthesis. CCD7 cleaves 9-*cis*-β-carotene formed by the *cis*-*trans*-isomerase D27 (Alder et al., 2012; Bruno et al., 2014; Bruno and Al-Babili, 2016; Abuauf et al., 2018) to produce the apocarotenoids 9-*cis*-β-apo-10′-carotenal (C_27_) and β-ionone (C_13_). CCD8 catalyzes a combination of reactions converting 9-*cis*-β-apo-10′-carotenal into carlactone, the central intermediate in SL biosynthesis (Figure 1; Alder et al., 2012; Bruno et al., 2014; Bruno and Al-Babili, 2016; Abuauf et al., 2018). Thereby, it is assumed that CCD8 mediates isomerization, intramolecular rearrangement, and repeated oxygenation reactions (Alder et al., 2012; Bruno et al., 2017).

**Figure 2:**
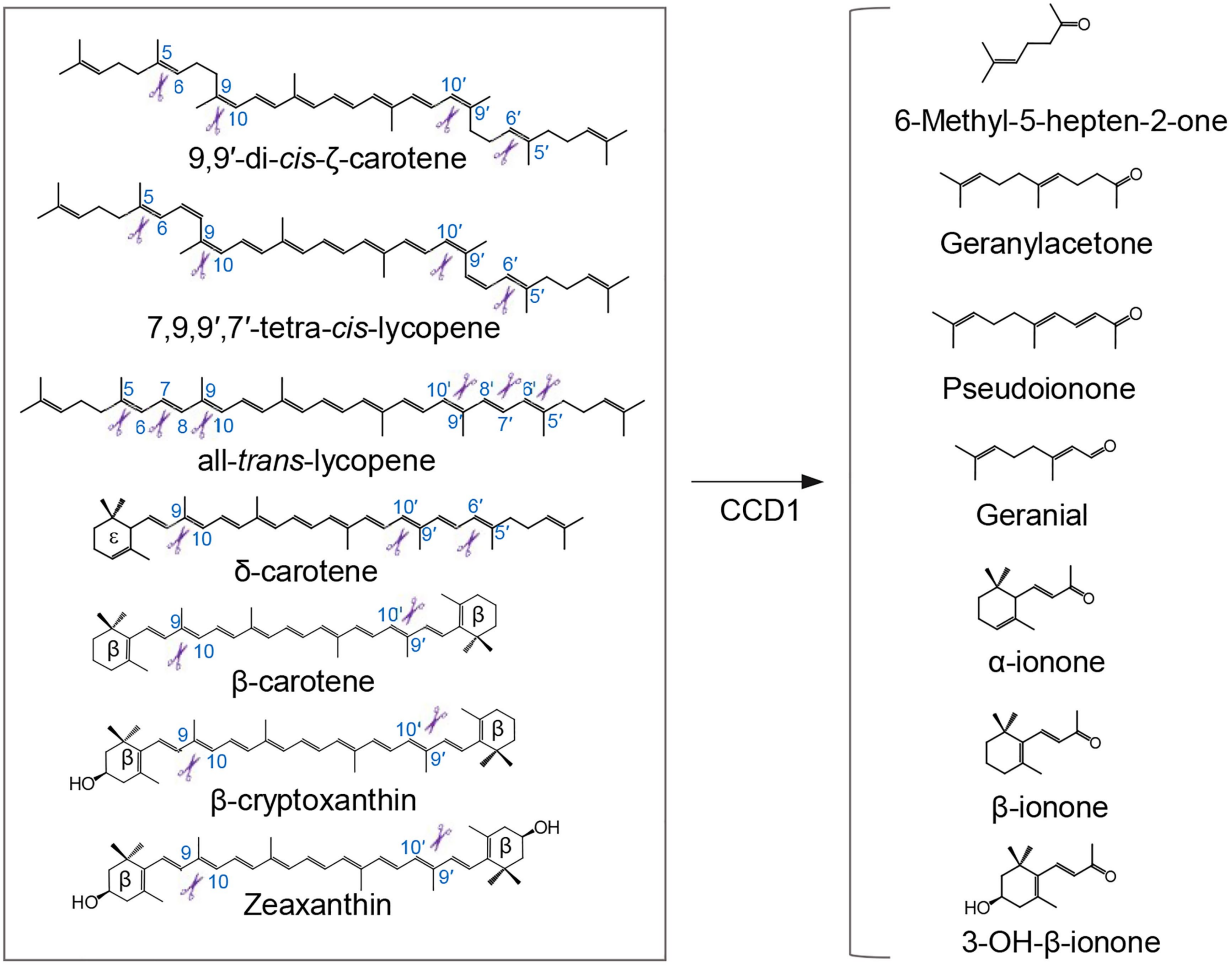
Cleavage of carotenoid substrates by CCD1 enzyme and examples of generated volatiles. Plant CCD1 enzymes can cleave linear, monocyclic, and bicyclic carotenoid and apocarotenoid substrates at different double bonds in *E. coli* and/or *in vitro*, yielding various apocarotenoid volatiles, in addition to a plentitude of apocarotenoid dialdehydes (not shown). Scissors indicate the double bond positions cleaved by CCD1.

Plant CCD1 enzymes, characterized by their relaxed cleavage site and substrate specificity, cleave linear, monocyclic, and bicyclic carotenoid and apocarotenoid substrates at different double bonds (Figure 2), yielding various apocarotenoid aldehydes and ketones with different chain lengths (Schwartz et al., 2001; Vogel et al., 2008; Ilg et al., 2009, 2014). Some CCD1 cleavage products are volatile compounds that contribute to the aroma and flavor of fruits and flowers of important horticulture crops. For example, CCD1-catalyzed C9-C10 and/or C9′-C10′cleavage of carotenoids forms C_14_ dialdehyde and C_13_ ketone products (Figure 2), including β-ionone, β-ionophore, α-ionone, pseudoionone, and geranylacetone, which are flavor and fragrance volatiles in fruits or flowers of many different plant species, such as tomato (Simkin et al., 2004a), grape (Mathieu et al., 2005), melon (Ibdah et al., 2006), and petunia (Simkin et al., 2004b). CCD1 was also shown to cleave lycopene at the C5-C6 and/or C5′-C6′ position to yield MHO (Figure 2), an important aroma compound in tomato (Vogel et al., 2008). Additionally, the rice CCD1 enzyme showed an *in vitro* enzymatic activity that targets the C7-C8 bond of lycopene (Figure 2), generating a C_10_ flavor compound, geranial (Ilg et al., 2009). However, CCD1 enzymes are localized to cytoplasm, therefore they seem to cleave already destructed carotenoids transported to cytoplasm (i.e., apocarotenoids) rather than carotenoid substrates in plastid (Ilg et al., 2009, 2010). Consistent with this speculation, overexpression of rice CCD1 in Golden Rice did not lead to significant decrease of the carotenoid content in endosperm (Ilg et al., 2010).

**Figure 3:**
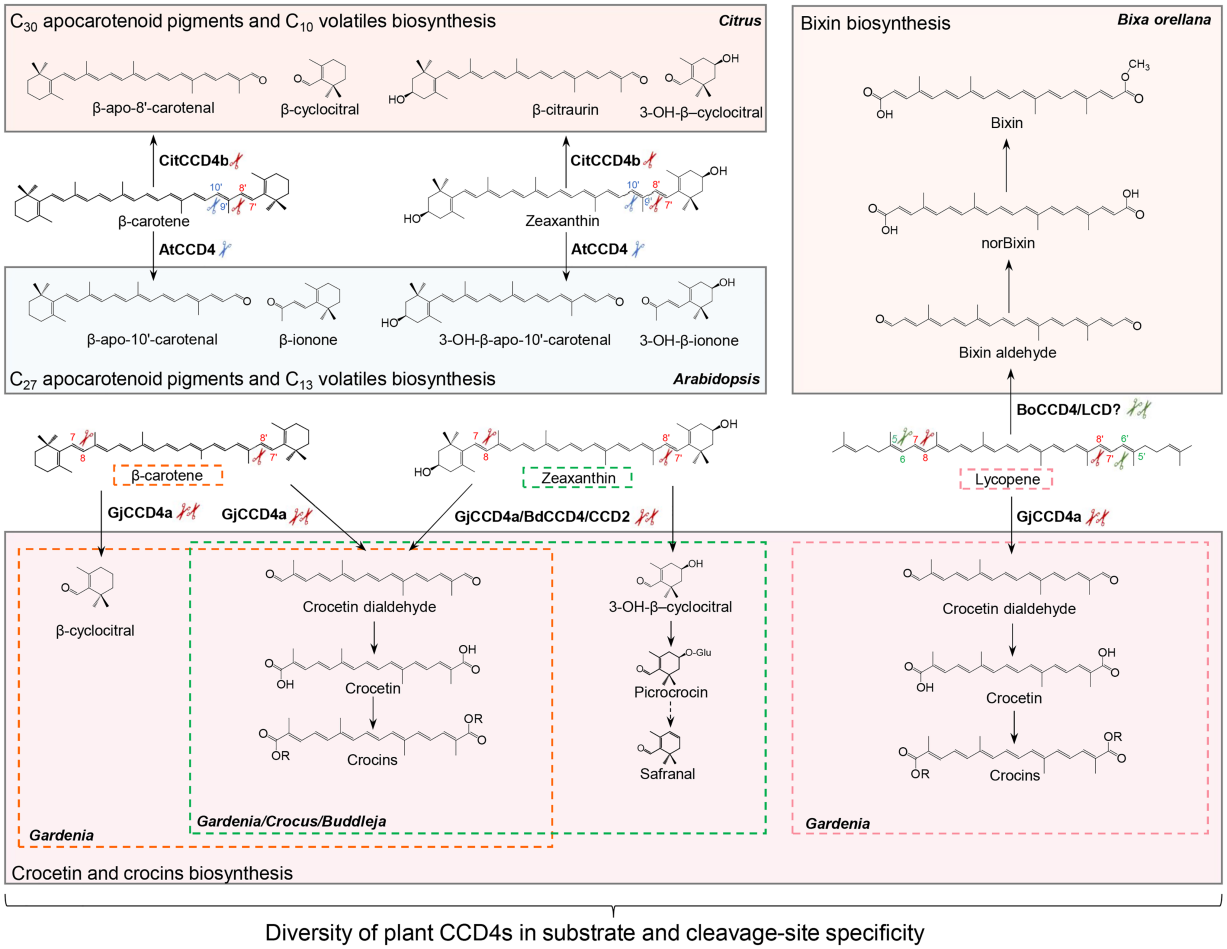
Diversity of carotenoid cleavage dioxygenase 4 (CCD4) cleavage reactions. Names of apocarotenoids are shown in black. Names of carotenoid cleavage dioxygenases are in depicted black boldface. Scissors in different color indicate the positions cleaved by CCD4 or CCD2 enzymes from different plant species. Abbreviations: CitCCD4b, *Citrus* CCD4b; AtCCD4, *Arabidopsis thaliana* CCD4; GjCCD4a, *Gardenia jasminoides* CCD4a; BdCCD4, *Buddleja davidii* CCD4, CsCCD2, *Crocus sativus* CCD2; and BoCCD4/LCD, *Bixa orellana* CCD4/LCD.

CCD2, a specific CCD type that is restricted to the *Crocus* and *Freesia* genus of the *Iridaceae* family, is closely related to the cytoplasmic CCD1 subfamily, although it is localized in plastids (Frusciante et al., 2014; Ahrazem et al., 2016b; Fang et al., 2020). Crocus CCD2 was shown to cleave the C7-C8 and C7′-C8′ double bonds of zeaxanthin to form crocetin dialdehyde (C_20_) and 3-OH-β-cyclocitral (C_10_; Frusciante et al., 2014; Ahrazem et al., 2016b), which are subsequently converted to crocins and safranal (Figure 3), respectively, contributing to the color and aroma of saffron stigma (Demurtas et al., 2018; Diretto et al., 2019).

Carotenoid cleavage dioxygenase 4s are a further type of plastid-localized CCDs. They are involved in the production of apocarotenoid volatiles and their activity determines carotenoid content and apocarotenoid pigments accumulation (Ohmiya et al., 2006; Campbell et al., 2010; Brandi et al., 2011; Gonzalez-Jorge et al., 2013; Zhang et al., 2015). CCD4 enzymes from different plant species vary in their substrate specificity and regioselectivity (Figure 3; Mi and Al-Babili, 2019). In Arabidopsis and potato, CCD4 was shown to catalyze the cleavage of bicyclic carotenoids at the C9-C10 or C9′-C10′ double bond to produce C_13_ volatiles, and C_27_ apocarotenoids that are supposed to be further degraded to smaller metabolites (Figure 3; Bruno et al., 2016). This enzymatic activity of CCD4 decreases carotenoid content in various tissues of plant species, including potato tubers, Chrysanthemum and Brassica flowers, Japanese morning glory petals, peach fruits, and Arabidopsis seeds (Ohmiya et al., 2006; Campbell et al., 2010; Brandi et al., 2011; Falchi et al., 2013; Gonzalez-Jorge et al., 2013; Zhang et al., 2015; Watanabe et al., 2018; Han et al., 2019a). In contrast, the *Citrus* CCD4b targets at a different site, i.e., the C7-C8 or C7′-C8′ double bond, of β-carotene, β-cryptoxanthin, and zeaxanthin to form C_30_ apocarotenoid pigments, such as β-citraurin, together with C_10_ volatiles, such as β-CC in citrus peel (Figure 3; Ma et al., 2013; Rodrigo et al., 2013; Zheng et al., 2015, 2019, 2021). It is believed that *Citrus* CCD4b is also involved in the production of β-citraurinene and β-citraurol, two further *Citrus* fruit-specific C_30_ apocarotenoid pigments in citrus peels (Zheng et al., 2021). Indeed, C_30_ apocarotenoids are the main long-chain apocarotenoids in *CCD4b*-overexpressing citrus callus, reinforcing the major roles of CCD4b in the biosynthesis of C_30_ pigments in citrus peel (Zheng et al., 2019, 2021). The BdCCD4.1 and BdCCD4.3 from *Buddleja davidii*, showing the same enzymatic activity of crocus CCD2, cleave C7-C8, and C7′-C8′ double bonds of zeaxanthin *in vitro* and *in vivo*, to form crocetin dialdehyde and 3-hydroxy-β-cyclocitral (Ahrazem et al., 2017). A recent study reported another CCD4 type from *Gardenia jasminoides* that cleaves several substrates, including β-carotene, zeaxanthin, and lycopene, at both C7-C8 and C7′-C8′ double bonds, and is responsible for the production of crocetin and crocins in gardenia fruit (Figure 3; Xu et al., 2020b).

Zaxinone synthase (ZAS), also referred to as CCD10, is a new CCD-type that is missing in Arabidopsis and which has been recently unraveled in rice and maize (Wang et al., 2019a; Zhong et al., 2020). The rice ZAS was shown to cleave the 3-ΟΗ-apocarotenoids with different chain lengths, such as C_30_ all-*trans*-3-OH-apo-8′-carotenal, C_27_ all-*trans*-3-OH-apo-10′-carotenal, and C_25_ all-*trans*-3-OH-apo-12′-carotenal, at the C13-C14 double bond to yield a C_18_-ketone product, zaxinone, that is characterized as a signaling molecule regulating plant growth and development (Wang et al., 2019a). It seems likely that the presence of this enzyme correlates with the ability to establish mycorrhizal symbiosis, as non-mycorrhizal plants, such as *Brassica* species, do not contain this type of CCDs.

### Unspecific Oxidative Cleavage Processes in the Formation of Apocarotenoids

There are other enzymes mediating carotenoid cleavage, besides CCDs. For instance, lipoxygenases (LOXs) use carotenoids as co-substrates, however, resulting in unspecific oxidative cleavage of carotenoids. The fatty acid peroxyl radicals produced by LOXs enzymatic peroxidation of polyunsaturated fatty acids, such as linolenic, arachidonic, and linoleic acid, can attack carotenoids, leading to their degradation, as was shown *in vitro* (Jarén-Galán and Mínguez-Mosquera, 1999). Disruption and downregulation of *LOX* genes reduced carotenoid degradation during pasta processing and decreased the degradation of carotenoids in Golden Rice during storage, respectively (Carrera et al., 2007; Gayen et al., 2015), reinforcing the role of LOXs in carotenoid oxidative cleavage. Recently, by using pan-genome analysis, quantitative trait locus mapping and functional analysis, Gao et al. found that one tomato LOX (referred to as TomLoxC) is responsible for the production of apocarotenoid volatiles, including β-CC and β-ionone, in tomato fruits (Gao et al., 2019).

In addition to unspecific enzymatic degradation processes, carotenoids are subjected to unspecific non-enzymatic destructive oxidation triggered by reactive oxygen species (ROS; Ramel et al., 2012; Hou et al., 2016), which initiates metabolic pathways yielding various signaling molecules and/or volatile compounds. For example, singlet oxygen formed in chloroplasts, particularly at photosystem (PS) II under high-light condition, attacks all-*trans* β-carotene, giving rise to the formation of various apocarotenoid aldehydes, ketones, endoperoxides, epoxides, and lactones (Stratton et al., 1993; Hasegawa et al., 2004; Fiedor et al., 2005; Yamauchi et al., 2014), including the growth regulator and signaling molecule, β-CC (Ramel et al., 2012; Dickinson et al., 2019). It is assumed that linear volatile apocarotenoids, such as MHO, citral, geranylacetone, pseudoionone, and farnesylacetone, can also be generated through the unspecific oxidative degradation of carotenoid precursors, besides the possible involvement of CCD1 enzymes (Simkin et al., 2004a; Moreno et al., 2021).

## Molecular Regulation of Apocarotenoid Biosynthesis

The production of plant apocarotenoids varies depending on developmental stage and tissue and is affected by environmental stimuli that lead to non-enzymatic cleavage of carotenoid. Due to their role as regulatory metabolites, the genesis of several apocarotenoids is tightly regulated at different levels, including transcriptional, post-translational, and epigenetic regulation.

### Transcriptional Regulation

Many studies have reported that some CCD genes show tissue/organ-specific expression pattern that also depends on growth/developmental stages and environmental conditions (García-Limones et al., 2008; Chiou et al., 2010; Gonzalez-Jorge et al., 2013; Yuan et al., 2015; Zheng et al., 2019). Several transcriptional regulators are known to regulate the expression of genes related to apocarotenoid production in flowers and fruits of horticulture crops. For instance, sweet osmanthus (*Osmanthus fragrans*) OfWRKY3 and OfERF61 transcription factors were shown to directly bind to the CAACA and W-box elements in *OfCCD4* promoter, which results in an increase of *OfCCD4* expression level (Han et al., 2016, 2019b). It is assumed that these two transcription factors are positive regulators of the biosynthesis of β-ionone, a key aromatic component in sweet osmanthus petals, which is formed through OfCCD4 cleavage reaction (Huang et al., 2009; Han et al., 2016, 2019b). In grapes, the expression level of *VvCCD4b* was shown to positively correlate with the content of several apocarotenoid volatiles, including MHO, β-ionone, and β-damascenone. *VvMADS4*, a MADS family transcription factor, directly binds to the promoter of *VvCCD4b* and represses its expression (Meng et al., 2020). Transcription factors that activate apocarotenoid biosynthetic genes, in some cases, also increase the expression of carotenoid biosynthetic genes responsible for the production of the carotenoid substrates. For instance, the citrus CsMADS6 was shown to directly bind to and to activate the promoters of citrus *CCD1*, as well as those of carotenoid biosynthetic genes, such as *LCYb1* (*Lycopene β-cyclase 1*), *PSY* (*phytoene synthase*), and *PDS* (*phytoene desaturase*; Lu et al., 2018). Very recently, Zhu et al. (2021) found that citrus CsERF061 enhances the expression levels of *CCD1*, *NCED3*, and *CCD4* by direct binding to their promoters and to those of the seven carotenoid biosynthetic genes, *PSY1*, *PDS*, *carotene isomerase* (*CRTISO*), *LCYB1*, *LCYB2*, *β-carotene hydroxylase* (*BCH*), and *zeaxanthin epoxidase* (*ZEP*), indicating the involvement of CsERF061 in a multi-target regulation of carotenoid and apocarotenoid metabolism.

The CsULT1 Ultrapetala transcription factor, identified in crocus, was found to positively regulate the gene expression of *CCD2* and *CCD4b*, as well as of *PSY*, *PDS*, and *BCH* genes (Ashraf et al., 2015).

Unspecific enzymatic cleavage of carotenoids is also regulated at transcriptional levels. In tomato, overexpression of *SlMYB75*, a MYB-type transcription factor, resulted in significant increase in the expression level of *LOXC* gene that is involved in unspecific enzymatic production of volatile apocarotenoids (e.g., β-CC). Yeast one-hybrid assay revealed that SlMYB75 can directly bind to MYBPZM and MYBPLANT *cis*-elements in *LOXC* promoter. Further dual-luciferase assays proved the role of SlMYB75 in activating the promoters of the *LOXC* (Jian et al., 2019).

It should be mentioned that although these transcription factors have been shown to regulate the expression levels of carotenoid and/or apocarotenoid biosynthetic genes, their effects on the apocarotenoid content and composition in target tissues are still poorly understood. Further quantification of apocarotenoid pigments and/or volatiles by LC-MS/GC-MS profiling should be performed in plants overexpressing or lacking these transcription factors, to confirm their contribution to apocarotenoid production.

As it can be expected, apocarotenoid-derived hormones biosynthesis is also regulated by transcriptional factors in various tissues/organs. For example, basic pentacysteine 1 (BPC1) can bind to the promoter of *CCD7*, and transient overexpression of *BPC1* repressed *CCD7’s* promoter activity in roots of *Malus baccata* (Yue et al., 2015). The CDF4 transcription factor from Arabidopsis, a DOF family protein, is a positive regulator of ABA biosynthesis and leaf senescence, which increases the expression of *NCED2* and *NCED3* by directly binding to their promotors (Xu et al., 2020a). A NAC transcription factor, ATAF1, from Arabidopsis was shown to bind to the promoter of *NCED3 in vivo* and to activate its expression, which resulted in increased ABA levels. Moreover, a rice *NAC* transcription factor OsNAC2 also upregulates the expression of *OsNCED3* as well as *OsZEP1* and represses the expression of the ABA catabolic gene *OsABA8ox1*, which results in an increase of ABA levels and accelerated leaf senescence. In peach fruit, the ethylene response transcription factor PpERF3 was found to bind to the promoters of *PpNCED2/3* genes and to promote their expression, which results in an increased ABA content during fruit ripening (Wang et al., 2019b). In *Citrus reticulata* fruit, the R2R3-MYB transcriptional factor CrMYB68 is a negative regulator of *CrBCH2* and *CrNCED5* gene expression, contributing to the delay in the biosynthesis of ABA in “Green Ougan,” a stay-green mutant of *C. reticulata* cv. Suavissima (Zhu et al., 2017). CrMYB68 also interacts with a novel NAC transcription factor, CrNAC036, during binding to the promoter of *CrNCED5*, causing a synergetic effect in inhibiting ABA biosynthesis in *C. reticulata* fruits (Zhu et al., 2020). Very recently, a citrus homeodomain leucine zipper I transcription factor called CsHB5 was found to positively regulate ABA accumulation and senescence, by directly binding to the promoters of carotenoid and ABA biosynthetic genes, including *BCH* and *NCED2*, and activating their transcription (Zhang et al., 2021). In Arabidopsis seeds, the basic helix-loop-helix transcription factor bHLH57 was shown to induce the expression of *NCED6* and *NCED9* through binding to the E-box (CANNTG)/G-box (CACGTG) motifs within their promoters, thereby leading to higher ABA levels that impose seed dormancy. In contrast, reversal of rdo5 (ODR1), a Homolog of Rice Seed Dormancy4, was shown to decrease seed dormancy by directly interacting with bHLH57 and inhibiting the bHLH57-mediated induction of *NCED6* and *NCED9* in the nucleus (Liu et al., 2020).

### Post-translational Regulation

Post-translational regulation *via* protein-protein interactions provides an alternative mechanism to regulate CCD enzymatic activity in plants, thereby regulating the content and composition of apocarotenoids. PGM48, a plastoglobule (PG)-localized metallopeptidase identified in Arabidopsis, was found to positively regulate leaf senescence (Bhuiyan et al., 2016; Bhuiyan and van Wijk, 2017). PGM48 is the only protease present in PG proteomes. The protein level of PGM48 increased significantly during leaf senescence, whereas PG-localized CCD4 decreased. Indeed, overexpression of PGM48 in *Arabidopsis* dramatically reduced protein levels of CCD4. Moreover, it was shown that PGM48 can directly interact with CCD4, suggesting that CCD4 could be a substrate of PGM48 (Bhuiyan et al., 2016; Bhuiyan and van Wijk, 2017). The direct interaction and co-localization of PGM48 and CCD4 provided new insights into post-translational regulation of CCD enzymes; however, the role of PGM48 on the production of apocarotenoids is still elusive. Further apocarotenoids profiling and comprehensive characterization of the peptidase activity of PGM48 are still needed (Bhuiyan et al., 2016; Bhuiyan and van Wijk, 2017).

Plastid-localized ORANGE (OR), a DnaJ cysteine-rich protein, was found to act as a post-translational regulator of the protein level and enzymatic activity of PSY and a determinant of carotenoid accumulation (Zhou et al., 2015; Park et al., 2016; Chayut et al., 2017). Recent evidence showed that OR protein of sweet potato (IbOR) can interact not only with the known target PSY, but also with a new target, CCD4, suggesting that IbOR might also affect carotenoid cleavage as an important factor in carotenoid homeostasis (Park et al., 2020). However, the impact of this interaction on CCD4 protein level or activity is still elusive.

### Epigenetic Modification

Epigenetic regulation is a further way deployed by plants to regulate apocarotenoid biosynthesis. For example, methylation analysis of *Oncidium OgCCD1* promoters of white-colored “Jade” and yellow-colored “Gower Ramsey” (GR) cultivars showed that a high level of DNA methylation in GR *OgCCD1* promoter, which is consistent with a silent *OgCCD1* and higher content of carotenoids in yellow floral tissues of GR (Chiou et al., 2010). In citrus callus, treatment with the DNA methyltransferase inhibitor 5-azacytidine resulted in a dramatic decrease of carotenoid content along with significant upregulation of *CpCCD1* gene (Xu et al., 2017). However, in this study, the tested promoter regions of *CpCCD1* were not demethylated by the 5-azacytidine effect, suggesting that methylation changes may indirectly regulate expression of *CpCCD1* (Xu et al., 2017). Chiou et al. (2010), found that the *Oncidium OgCCD1* promoter region of yellow-colored GR cultivar showed a higher level of DNA methylation than that in “Jade,” which may result in the downregulation of *OgCCD1* in GR, thus leading to less conversion of carotenoids to colorless apocarotenoid in floral tissue. Moreover, a METHYLTRANSFERASE1 (SlMET1) acts as facilitator for *SlNCED* gene expression *via* directly altering methylation levels of *SlNCED* promoter region, thereby affecting ABA production in tomato epimutant *Colorless non*-*ripening* fruits (Yao et al., 2020).

## Genetic Insights Into Natural Variation of Apocarotenoid

Many crop plants, especially horticulture crops, show large variation in quantity and types of apocarotenoids among different species and/or different natural accessions of a single species. For instance, different papaya accessions display big natural variation in accumulating MHO and β-ionone in fruits (Jing et al., 2015). Farnesyl acetone, α-ionone, and β-ionone show also high natural variation among orange-colored “Nairobi” and “Rothild,” yellow-colored “Yellowstone,” purple “Purple Haze,” and white “Creme de Lite” carrot accessions (Yahyaa et al., 2013). As direct non-enzymatic degradation is supposed to be a major source of apocarotenoids, different concentration of carotenoids may be the reason for the diversity in apocarotenoid levels; however, more and more studies reported that some genetic factors can also determine the apocarotenoid variation, independent of carotenoid levels. For example, in citrus, α-ionone, geranial, and β-ionone are common in “Temple” hybrid mandarin fruit, but cannot be detected in “Murcott” hybrid that even contains significantly higher carotenoid amounts (Yu et al., 2015), In apricot fruit, β-ionone and dihydro-β-ionone are considered as two of the major aroma compounds for fruit quality by odor activity values (OVA) method. Contents of β-ionone and dihydro-β-ionone were found to be significantly higher in pulps of white apricot cultivars, such as “Luntaixiaobaixing” and “Baixing,” than in those of the yellow “Hongyuxing” and “Danxing” cultivars that even accumulate higher carotenoid content (Xi et al., 2016). This finding suggests that the variation of apocarotenoid content in different apricot species is also genetically controlled, other than only determined by the concentration of the carotenoid precursors.

**Figure 4:**
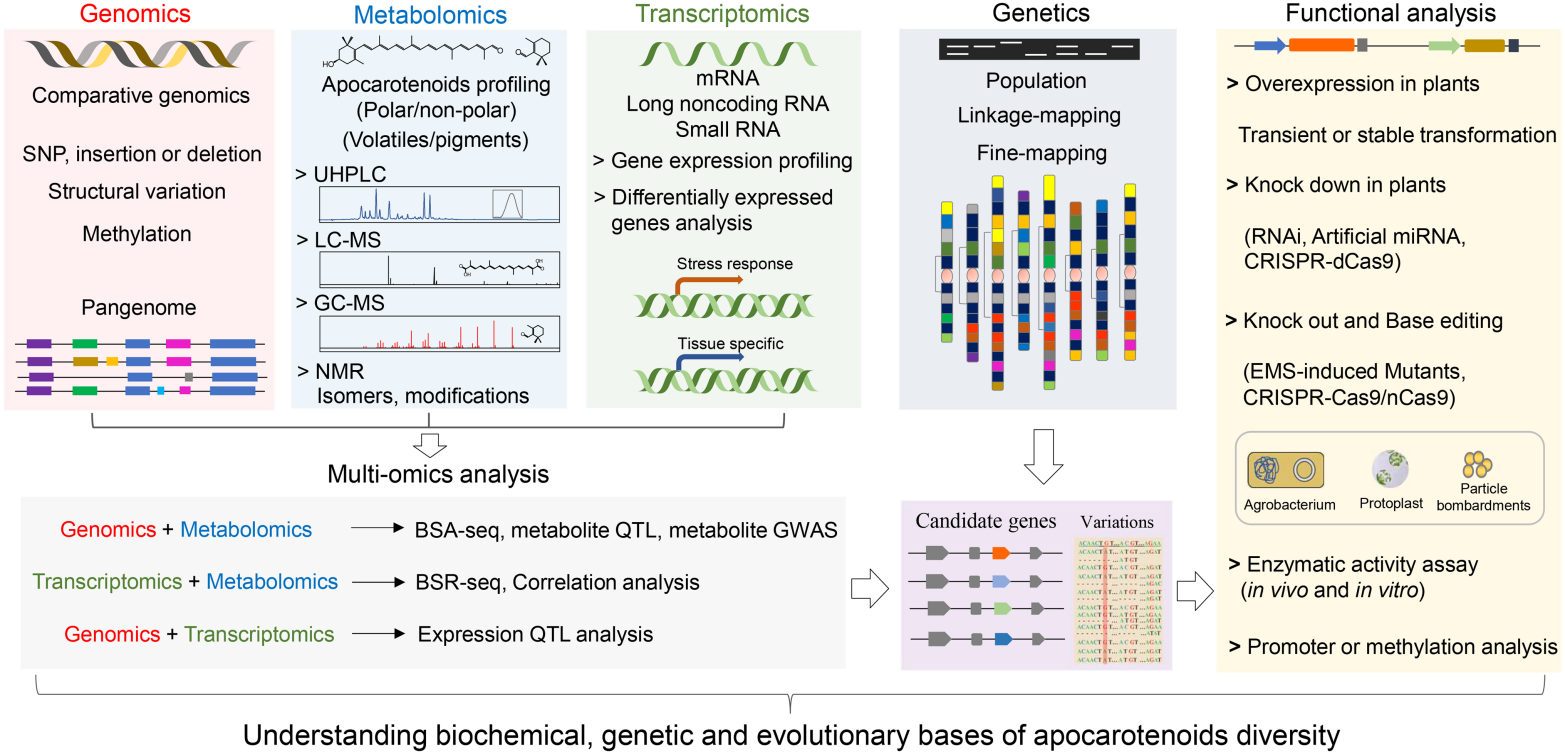
A schematic workflow for multi-omics strategy coupled with genetic and functional analysis to elucidate the molecular mechanisms underlying apocarotenoid variation. The multi-omics strategy combines genomics, transcriptomics, and metabolomics approaches. Functional analysis includes overexpression, knockdown, and knockout of candidate genes, which can be performed by using Agrobacterium-, protoplast-, or particle bombardments-mediated transformation strategies. For structural genes, such as *CCD* genes, evaluation of the activity of encoded enzymes (*in vivo* and *in vitro*) can be performed to confirm the functional variation. Promoter and methylation analysis are usually conducted to explore genetic bases underlying expression variation of candidate genes. GWAS, genome-wide association study; QTL, quantitative trait locus; eQTL, expression QTL; and mQTL, metabolites QTL. BSA-Seq, bulked-segregant analysis (BSA) coupled to whole-genome sequencing. BSR-Seq, BSA coupled to RNA-seq (RNA-sequencing).

Technological advancements in high-throughput sequencing, metabolomics, and data mining have enabled the integration of various omics datasets, such as genomics, transcriptomics, and metabolomics (Figure 4). The multi-omics strategy coupled with genetic and functional analysis has facilitated exploiting the natural variation of apocarotenoid pigments and volatiles, which paves the way new discoveries in metabolism, function, and evolution evolutionary of plant apocarotenoids.

For instance, Gao et al. (2019) constructed a tomato pan-genome by using genome sequence datasets of 725 tomato accessions. Comparative analyses using this constructed tomato pan-genome identified a rare allele in cultivated tomatoes defined by promoter variation, i.e., a ~ 4-kb substitution in the promoter region, of *TomLoxC* gene encoding a 13-lipoxygenase. Further RNA-Seq data analysis showed that accessions containing both this rare allele captured by pan-genome and another allele present in the “Heinz 1706” genome exhibit significantly higher expression levels of *TomLoxC* gene than that in other accessions. Further metabolites, QTL and gene expression analysis provided strong evidence that *TomLoxC* variation is a genetic control for natural variation in the accumulation of nine apocarotenoid volatiles common in tomato fruits and might additionally play an important role in apocarotenoid volatiles biosynthesis. The levels of specific apocarotenoid volatiles were also significantly decreased in transgenic tomato fruits with reduced expression of *TomLoxC* and in two knock-out Arabidopsis mutants of *AtLOX2*, the closest *TomLoxC* homolog. These experiments further confirm that *LOX* is a genetic determinant for natural variation of apocarotenoid formation and also reinforce the significance of alternative apocarotenoid biosynthesis route *via* unspecific oxidation process, besides CCD-mediated cleavage reaction. Apocarotenoid volatiles are important quality trait for tomato fruit and flavor (Tieman et al., 2017; Gao et al., 2019). However, this *TomLoxC* promoter allele captured by pan-genome displays strong negative selection during domestications. This may be due to the primary focus of modern breeding on tomato yield and on resistance to stresses and long shelf-life, which did not consider aroma quality traits.

In addition to volatiles, several recent studies performed apocarotenoid pigments profiling in fruits and related natural variation in some important horticulture crops, such as *Citrus* and *Capsicum* species. Citrus fruits show peel color variation among different cultivars. Besides carotenoid content and composition, the production of C_30_ apocarotenoid pigments, such as β-citraurin, is also critical for peel pigmentation of citrus mature fruit (Ma et al., 2013; Rodrigo et al., 2013; Zheng et al., 2015, 2019, 2021). Indeed, the content of C_30_ apocarotenoid pigments, i.e., β-citraurin and β-citraurinene, and their ratio to total carotenoids in citrus peel showed the high correlation with the color index of red-peeled progenies in a F_1_ pseudo-testcross population derived from a cross between a red-peeled *Citrus reticulata* and a yellow-peeled *Poncirus trifoliata* (Zheng et al., 2019). By using mQTL, eQTL, and BSR-seq analysis, this study showed that a 5′ *cis*-regulatory mutation of *Citrus CCD4b* (*CitCCD4b*) is a major genetic determinant of natural variation in the accumulation of the C_30_ pigments β-citraurin and β-citraurinene. This study demonstrated that CitCCD4b enzyme is involved in the biosynthesis of β-citraurinene, which indicates a novel reaction in modification of aldehyde group of β-citraurin. The presence of a specific SNP in the MITE of *CitCCD4b* promoter is strongly correlated with the high expression level of *CitCCD4b* among progenies of pseudo-testcross population as well as within 115 different citrus accessions, resulting in the high-accumulation of C_30_ apocarotenoid pigments responsible for the red coloration of citrus peel. A recent study also found that a red-peeled mutant of “Huyou” (*Citrus changshanensis*) contains much higher content of the C_30_ apocarotenoid β-citraurin, accompanied by 100 times higher expression level of *CitCCD4b*, compared to yellow-peeled ordinary “Huyou” (Luan et al., 2020b), which also correlated with the above-mentioned putative enhancer SNP in the *CitCCD4b* promoter (Zheng et al., 2019; Luan et al., 2020a). Very recently, it was found that the higher transcript level of *CitCCD4b* in red peel of F1 hybrids also accompanied the higher content of the two C_10_ apocarotenoid volatiles, β-CC and 3-OH-β-cyclocitral, in comparison to that in the yellow peel of three other progenies (Zheng et al., 2021). This result suggests a dual role of *CitCCD4b* expression variation in the natural variation of both apocarotenoid pigments and volatiles among different citrus species and in fruit pigmentation and aroma formation.

However, in other crops, such as peach and *Brassica* species, mutations in coding sequence of CCD4 enzyme enhanced the carotenoid pigments in tissues/organs, due to the capability of this enzyme in cleaving colorful carotenoids into C_27_ apocarotenoids that are catabolized into colorless smaller metabolites (Falchi et al., 2013; Mi and Al-Babili, 2019). Recent Studies demonstrated that CRISPR/Cas9-mediated disruption of CCD4 gene in *Ipomoea nil* and carrot resulted in a remarkable enhancement of carotenoids and led to pale-yellow phenotype in petal of *I. nil* and yellow phenotype in the taproot of a white-colored carrot variety, respectively (Watanabe et al., 2018; Li et al., 2021). The corresponding CCD4 enzymes have also been reported to be responsible for the formation of apocarotenoid volatiles in tissues/organs, such as in peach fruit (Brandi et al., 2011). The crucial step of future research is to determine whether the natural genetic variation of such *CCD4*s is also linked to the diversity of apocarotenoid volatiles among different accessions, which may be utilized in molecular breeding for improvement of fruit/vegetable flavor.

Apart from apocarotenoid pigments and volatiles, apocarotenoid-derived hormones also show natural variation among different accessions. For example, at low water potential, *Arabidopsis* accession “Shahdara” (Sha) showed less accumulation of ABA than that in “Columbia” (Col) or “Landsberg *erecta*” (Ler). Genetic analysis of Ler × Sha recombinant inbred lines (RILs) and complementation experiments demonstrated that *NCED3* variation is responsible for the difference in ABA accumulation between Sha and Ler accession. Further sequence analysis, site-directed mutagenesis and structural observations revealed that nonsynonymous substitutions of *NCED3* coding sequence found in Sha are associated with decreased ABA accumulation and altered NCED3 post-translational processing, which may affect NCED3 activity (Kalladan et al., 2019). The rice cultivars “Bala” and “Azucena” showed large difference in SL biosynthesis that is responsible for their degree of tillering and distinct susceptibility to Striga infection (Cardoso et al., 2014). By using a “Bala” × “Azucena” F6 RIL population, Cardoso et al. (2014) identified a major QTL, a rearrangement of a 51- to 59-kbp stretch between 28.9 and 29.0 Mbp of chromosome 1, which explains most of the variation in the SL contents of rice exudates. This genomic rearrangement leads to deletion of two *AtMAX1* orthologs and is associated with low SL contents in 367 rice cultivars.

## Concluding Remarks and Future Outlook

Apocarotenoids are not just carotenoid degradation products but play important functions as antioxidants, pharmaceuticals, scents, and pigments, beside their role as precursors of plant hormones, signaling molecules, and growth regulators. In this review, we have briefly introduced the significance of apocarotenoid compounds for human health and their biological functions that are critical for plant development, biotic interactions, and stress response and provided an update on recently identified biosynthetic pathways and their regulation. More and more studies have shown that exogenous application of apocarotenoids is able to affect plant growth and development, as well as both abiotic and biotic stress resistance in many plants, which uncovered their biological function as regulatory metabolites. Nevertheless, the correlation between natural variation in the content of these apocarotenoids and plant phenotypes remains elusive. It is still unknown why apocarotenoids with slightly different structures have different activities and diverse functions in plants and some apocarotenoids, i.e., zaxinone, exerts contradictory effects depending on plant species. We proposed that the exploration of biochemical and genetic reasons of apocarotenoid metabolic diversity/variation could accelerate exploring currently unknown/unclear bioactive apocarotenoids pathway that could help understand their contributions to crops end-phenotypes, as well as their regulation and modifications that are still poorly understood. To date, the progress in determining natural genetic variation mechanisms and its molecular background is limited to only few apocarotenoid volatiles and pigments, while that of other important apocarotenoids, such as crocins, bixin, zaxinone, and anchorene, and apocarotenoid-derived phytohormones, is still poorly characterized. The rapid development of next-generation sequencing strategies provides reliable reference genomic sequences that are now available for most crop plants. We anticipate that mass spectrometry apocarotenoids profiling coupled with multi-omics strategies, including quantitative trait locus (QTL) mapping, genome-wide association (GWA), and RNA-seq analysis, will pave the way for uncovering new QTLs for apocarotenoid metabolite traits and identifying their underlying genes in various important crops (Figure 4). Advancement of knowledge in this area will enable to identify DNA marker-apocarotenoid traits associations for marker-assisted improvement of crops agronomic traits, such as aroma and color of fruits and flowers, related to apocarotenoid content, but also provide new metabolic engineering and/or CRISPR editing target for improvements of high-value beneficial apocarotenoids in crops in a low-cost, efficient, stable, and environment-friendly manner, to satisfy increasing demands for better crop quality in the future.

## Author Contributions

XZ organized and drafted the manuscript with substantial input from YY and designed and prepared the figures with input from YY. YY and XZ prepared the table. SA-B supervised the writing of the review and was involved in its further editing and revision. All authors contributed to the article and approved the submitted version.

## Funding

This work was supported by baseline funding and Competitive Research Grants (CRG 2017 and CRG 2020) given to SA-B from King Abdullah University of Science and Technology (KAUST).

## Conflict of Interest

The authors declare that the research was conducted in the absence of any commercial or financial relationships that could be construed as a potential conflict of interest.

The handling editor declared a past co-authorship with one of the authors SA-B.

## Publisher’s Note

All claims expressed in this article are solely those of the authors and do not necessarily represent those of their affiliated organizations, or those of the publisher, the editors and the reviewers. Any product that may be evaluated in this article, or claim that may be made by its manufacturer, is not guaranteed or endorsed by the publisher.
